# Associations Between Adoption Discounts, Length-of-Stay, and Adoption Rates of Dogs in an Open-Admission Municipal Animal Shelter in NSW, Australia

**DOI:** 10.3390/ani16020321

**Published:** 2026-01-21

**Authors:** Tianyang Qiu, Simone J. Maher, Evelyn Hall, Mark E. Westman

**Affiliations:** 1 Sydney School of Veterinary Science, The University of Sydney, Camperdown, NSW 2006, Australia; 2 Centre for Veterinary Education, The University of Sydney, Camperdown, NSW 2006, Australia; 3 PetSure Australia, Chatswood, NSW 2067, Australia; 4 The Sydney Institute for Infectious Diseases, The University of Sydney, Sydney, NSW 2006, Australia

**Keywords:** dogs, shelters, discount, animal welfare, adoption, length of stay, veterinary science

## Abstract

This study explored possible associations between adoption discounts, length-of-stay (LOS), and adoption rates for dogs at a municipal (council) shelter in New South Wales (NSW), Australia, over a one-year period (4 April 2023–3 April 2024). Data from 479 rehomed dogs and eight temporary promotional campaigns were analysed, considering the following factors: discount levels applied, breed group, body size, age group, coat colour, intake method (stray, privately surrendered, or seized), and return-to-shelter history after adoption. Dogs with ≥75% discount and 0–50% discount had a longer LOS compared to those without a discount (*p* < 0.001), likely because many discounted dogs already had a prolonged LOS prior to the campaign’s commencement. Other important LOS predictors included breed group (*p* < 0.001), body size (*p* < 0.001), age group (*p* = 0.004), and intake method (*p* < 0.001). Gundogs/hounds/terriers (purebred), and toy/non-sporting groups (both purebred and crossbred), small-sized dogs, seniors, puppies, and privately surrendered dogs had significantly lower LOS compared to their counterparts. However, when daily adoption rates were examined, temporary price-discounting campaigns resulted in substantially increased rehoming rates. In particular, Flash Sales (≤48 h) increased daily adoptions by 204% compared to non-campaign periods. One Flash Sale event resulted in higher daily adoption rates, but also significantly higher return rates compared to other temporary campaigns, highlighting a possible risk of impulse adoptions and necessitating future work with adopters to identify potentially problematic decision-making. Shelters should be aware that, on an individual level, factors other than price can be more important for potential adopters. On a broader level, temporary campaigns involving a reduced adoption price can increase overall adoption rates and therefore should be considered as part of any marketing exposure strategy for animal shelters.

## Simple Summary

Animal shelters worldwide sometimes organise promotional campaigns, including discounted adoption prices, often to relieve overpopulation when full capacity is approaching or reached. Reduced adoption prices are assumed to help animals be adopted faster. To investigate the relationship between adoption price discounts and length-of-stay (LOS) and adoption rates in dogs, data were collected over a one-year period from an open-admission municipal (council) shelter located in New South Wales (NSW), Australia, accepting stray, privately surrendered, and seized dogs. Breed group, body size, age group, coat colour, sex, intake method, and whether returned to the shelter after adoption were also considered as possible contributing factors. Discounted dogs waited longer to be adopted than full-price dogs. Gundogs, hounds, terriers, toy, and non-sporting breeds had the shortest LOS, while Staffordshire terriers had the longest LOS. Additionally, small-sized dogs, puppies, senior dogs, and privately surrendered animals had lower LOS and were more desirable to the adopting population. However, temporary price discounting overall had a positive effect on dog adoptions, with more dogs adopted per day during campaign periods than non-campaign periods. These findings demonstrate that, although other factors apart from cost are more important drivers for decision making on an individual level when it comes to adopting shelter dogs in Australia, temporary price reduction campaigns are still effective for increasing shelter exposure and higher adoption rates.

## 1. Introduction

The COVID-19 pandemic resulted in huge shifts in pet ownership globally, with a significant surge observed in both animal adoptions and animal surrenders [1]. According to a survey conducted by Animal Medicine Australia in July 2022, the number of dogs owned in Australia increased from 5.1 million to 6.4 million between 2019 and 2022, an increase of approximately 25% over a three-year period [2]. Worldwide, however, relinquishment of dogs and cats also increased during the COVID-19 pandemic, particularly of recently adopted animals, generating unprecedented challenges for animal shelters [1,3,4]. In Australia, many shelters became overwhelmed, leading to an urgent need for effective strategies to expedite animal adoptions, reduce length-of-stay (LOS), and prevent relinquishment of adopted animals.

In the state of New South Wales (NSW), Australia, the two primary animal welfare and management regulatory frameworks comprise the Prevention of Cruelty to Animals Act 1979 (NSW) and the Companion Animals Act 1998 (NSW) [5]. The Companion Animals Act 1998 (NSW) regulates the responsible ownership of companion animals (dogs and cats). Dogs and cats must be microchipped from 12 weeks-of-age and prior to sale or adoption, and they must be registered with the local city council by 6 months-of-age. Injured and stray dogs are accepted and managed by municipal shelters (operated by councils) or any authorised premises including animal welfare organisations (e.g., the Royal Society for the Prevention of Cruelty to Animals, Animal Welfare League NSW), or registered veterinary hospitals [6]. In Australia, breeders are the primary source for acquiring purebred (including ‘designer’) dogs, while shelters are the primary source of crossbred dogs [2].

Previous studies investigating animals in shelters awaiting rehoming have identified various animal signalment factors that may affect LOS, including age, size, sex, breed, coat colour, hair length, degree of socialisation, and behavioural evaluation findings [7,8,9,10,11,12,13]. Age is a consistently significant factor, with younger animals typically experiencing a shorter LOS than older animals [7,8,11,12,14]. With regard to body size, a study conducted in the Czech Republic reported that the smaller the dog, the shorter the LOS [12]. Another study from the USA suggested a similar pattern, except medium-sized dogs (not large dogs) had the longest LOS [8]. Sex can impact LOS, with male dogs staying longer in shelters than female dogs in the Czech Republic and Poland [11,12]. Breed is also considered an important factor for both dogs and cats, with purebred animals typically rehomed faster than non-purebred animals [11,15,16,17,18], and canine “fighting breeds” and guard breeds being regarded as less desirable by potential adopters and therefore generally more difficult to rehome [8,9]. In dogs, the removal of breed labelling from adoption profiles, photographs, and videos for pitbull-type breeds (a breed considered less desirable in the USA) resulted in increased adoption rates, lower euthanasia rates, and a reduced LOS [9]. A study conducted by Nakamura et al. in Australia found that, among the four breed groups investigated, Labrador Retrievers had the shortest LOS, followed sequentially by Jack Russell terriers, Staffordshire terriers, and Australian cattle dogs [19]. In cats, the mean LOS decreased significantly as the degree of socialisation increased, with the lowest LOS found in interactive cats, followed by approachable cats, and the highest LOS found in unapproachable cats [13]. Dogs at a New York shelter that exhibited dangerous behaviour during their evaluation had a longer LOS compared to dogs that did not display any dangerous behaviours [10].

Other non-animal signalment factors that might affect LOS have also been investigated, including the inclusion of adoption indemnity waivers and the narrative voice and descriptive texts used in adoption profiles [19,20,21]. In Australia, cats adopted with indemnity waivers (medical and/or behavioural) had a longer LOS than those adopted without waivers [20]. In the UK, cats with a description written in the third person had a shorter LOS compared to cats with adoption profiles written in the first person [21]. Australian cattle dogs described as “active” in their online adoption profile had a shorter LOS, while Staffordshire Bull terriers, Jack Russell terriers, and Labrador Retrievers had a shorter LOS when described as “gentle” and “quiet” [19].

Finance is also considered a major consideration and a potential barrier to pet ownership, with both current pet owners and non-owners in Australia highly concerned with initial and ongoing costs of caring for a pet [2]. Whether this is a factor for potential dog adopters in Australia is unknown. The primary aim of this study, therefore, was to identify any association between different levels of temporary discounts and the LOS of dogs rehomed by an open-admission municipal (council) shelter located in Sydney, NSW, Australia, that adopts a hedonic price model described by Reese et al. [22]. To the best of our knowledge, this is the first study to investigate a possible association between reduced adoption fees and LOS, while also considering animal signalment variables that might have affected LOS. A secondary aim of the study was to investigate daily adoption rates during temporary campaigns (when price discounting was applied) and non-campaign periods at the shelter. Addressing these research gaps will contribute significantly to the development of effective, evidence-based pricing strategies for animal shelters in Australia and overseas.

## 2. Materials and Methods

### 2.1. Shelter Intake and Assessment Procedures

Dogs are impounded at the shelter for three possible reasons: (i) stray dogs brought in by a member of the public or council ranger and held for the minimum statutory requirement (7 days if not microchipped, 14 days if microchipped), before ownership is transferred to the facility; (ii) privately surrendered by a legal owner (owner surrenders the animal to the shelter and pays a one-off relinquishment fee), or (iii) surrendered to the shelter via a council ranger or police officer (most commonly, dogs or their owners are involved in a complaint to the local council and the animal is effectively seized). Seized dogs are held at the shelter for a variable time, while council or police investigations are undertaken, before entering the adoption process.

All impounded dogs at the shelter receive an incoming physical examination within 24 h from an animal welfare officer (an authorised officer with a veterinary nursing background). If any medical abnormality is found, the dog is referred either to the council’s on-site veterinarian or to a contracted veterinary practice for further diagnostic workup. Healthy dogs are vaccinated during the physical examination with a subcutaneous injection of Protech^®^ C3 (Boehringer Ingelheim Animal Health Australia, Sydney, NSW, Australia), a live attenuated vaccine containing canine distemper virus, canine adenovirus type 2, and canine parvovirus. Additionally, Nobivac^®^ KC (MSD Animal Health, Sydney, NSW, Australia) is administered intranasally to prevent *Bordetella bronchiseptica* and canine parainfluenza virus infection. Simparica^®^Trio (Zoetis, Sydney, NSW, Australia) and Popantel^®^ (Zoetis, Sydney, NSW, Australia) are given orally to protect against heartworm, fleas, ticks, mites, and intestinal worms, including tapeworm.

After the facility has claimed ownership of the dog, a behavioural assessment is conducted by an experienced animal rehoming officer at the shelter. The behavioural assessment comprises two components: (i) an evaluation of in-kennel and lead-walking behaviours; and (ii) a 20 min assessment in the kennel and an exercise yard to determine the dog’s degree of sociability with people and other dogs, while also looking for any instances of inappropriate or aggressive behaviour. Following this two-part behavioural assessment, a dog is classified as suitable for adoption, not suitable for adoption, or undecided. If the assessment outcome is undecided, the animal is reassessed the following week for a final outcome to be determined. Once a dog has been deemed suitable for adoption, entire animals are neutered on-site at the shelter’s veterinary clinic or at a contracted veterinary practice before being made available for rehoming. Any findings noted during the medical and behavioural checks are disclosed to the potential adopters prior to adoption.

This retrospective study utilised data stored at the shelter in TechnologyOne Version 10.04.24.011 (Technology One Limited, Brisbane, QLD, Australia).

### 2.2. Description of Dataset

Dogs that were rehomed from the shelter between 4 April 2023 and 3 April 2024 were included in this study. A follow-up period of six months (4 April 2024 to 3 October 2024) was examined for returned animals from the previous 12-month adoption period. For the purposes of this study, a successful rehoming was defined as an adoption by a private individual within the designated 12-month period (4 April 2024 to 3 October 2024) when a monetary transaction occurred. Transfers to other organisations (e.g., rescue groups) were free of charge and therefore were not considered adoptions and excluded from analysis.

### 2.3. Length-of-Stay (LOS)

LOS was defined as the period from the date of impounding to the date of rehoming. The TechnologyOne database does not record the date that animals become available for adoption following any mandatory hold periods and the medical and behavioural assessments. Therefore, the LOS for stray dogs entering the facility included the mandatory holding period (7 days if the dog was not microchipped, 14 days if the dog was microchipped). The mandatory holding period did not apply to dogs that were privately surrendered by the previous owner directly to the facility. For seized dogs, the LOS included the holding period while council or police investigations were undertaken. For all three intake categories, LOS included the holding period while waiting for the medical and behavioural assessments and desexing (if required), prior to being made available for adoption.

### 2.4. Original Price and Actual Price Sold

The shelter employs a hedonic pricing strategy, categorising adoption prices based on the animal’s age, with adoption price decreasing with increasing age: under 4 years (most expensive), 4 to 7 years, and over 7 years (least expensive) (Table 1). The sale price for each animal rehomed during the study period was exported from the TechnologyOne cashier receipt system.

### 2.5. Adoption Discounts and Promotional Campaigns

The shelter hosted eight temporary promotional campaigns during the 12-month adoption period, ranging in duration from 1 to 31 days. For the purpose of assessing the effect of temporary price discounting on daily adoption rates, campaigns were categorised according to the length (time duration) of the campaign: Flash Sales (≤48 h), Short Campaigns (2–7 days), and Long Campaigns (>1 week). Additionally, during the study period there were permanent ongoing pricing offers for dogs that had stayed at the shelter for over 120 days (‘Long-term Special Pricing’) and for pensioners seeking to adopt (‘Pensioner Pricing’). Dogs rehomed as part of the two ongoing pricing categories were excluded from the current study because they are ongoing discounts instead of temporary discounts offered only during discrete promotional campaigns. Details on pricing, duration, eligible dogs, and other conditions in place during the eight campaigns and for the two ongoing pricing categories are provided in Table 2.

### 2.6. Discount Categories

The discount percentage was calculated as follows:

Discount percentage (%) = [(original price − actual price sold)/original price] × 100.

There were four price groups considered for analysis purposes: No discount (0%), 0–50% discount, 50–75% discount, and ≥75% discount.

### 2.7. Breed Categories

The breed details recorded on the NSW Pet Registry and/or Australian Central Animal Records online microchip databases were used for microchipped animals. If the animal was not microchipped or if the microchip information did not match the dog’s phenotypic traits, the primary breed was recorded by an experienced staff member using the phenotypic traits noted. For dogs with traits from multiple breeds, a secondary breed phenotype was recorded. The breed was then retrospectively assigned to one of seven breed groups (toys, terriers, gundogs, hounds, working dogs, utility, and non-sporting) by the primary author (T.Q.) based on the standards outlined by the Australian National Kennel Council [23]. This breed categorisation method was previously described by researchers in the USA [24] and adapted to the Australian context. In addition, American and English Staffordshire terriers were considered as a separate group to the terrier group because they represented most dogs housed at the shelter and awaiting rehoming. Each group was further subdivided into purebred and crossbred sub-groups, based on whether a secondary breed was recorded.

Breed groups with similar traits and functions were combined due to the small sample size among some of the breed groups. The final seven breed groups retained for analysis included purebred American/English Staffordshire terriers, crossbred American/English Staffordshire terriers, purebred utility and working dogs, crossbred utility and working dogs, other purebred dogs (gundog, hound, and terrier), other crossbred dogs (gundog, hound, and terrier), and toy and non-sporting dogs (both purebred and crossbred).

### 2.8. Body Size

Body size was assigned as either small, medium, or large following visual inspection by an animal welfare officer on the day of impoundment. These categories generally corresponded to estimated weights of <10 kg (small), 10–30 kg (medium), and >30 kg (large).

### 2.9. Age Categories

The age of incoming dogs was recorded by an animal welfare officer either by obtaining microchip details from the NSW Pet Registry and/or Australian Central Animal Records, or by estimation based on dentition and physical appearance. For statistical analysis, dogs were categorised into the following age categories: puppies (<6 months), young dogs (6 months to less than 12 months), young adults (1 year to less than 2 years), adults (2 years to less than 4 years), older adults (4 years to less than 8 years), and seniors (8 years or older).

### 2.10. Primary Coat Colour

The primary coat colour was assigned by an animal welfare officer during the intake process and recorded in TechnologyOne. The coat colour was then retrospectively categorised by the primary author (T.Q.) into seven groups: black, blue, brindle, tricolour, white, tan, and other (brown, chocolate, cream, fawn, gold, grey, red, or sable).

### 2.11. Sex

Since all dogs rehomed were desexed prior to becoming available for adoption (unless already neutered), the dogs were either male-neutered or female-neutered.

### 2.12. Animal Return

Dogs were classified as either not returned (i.e., still adopted), or returned-to-shelter if they were re-surrendered to the facility during the 18-month study period. For dogs returned to the shelter, only the first LOS were used for analysis (i.e., subsequent LOS were excluded).

The returned-to-shelter rate per discount category and per campaign/non-campaign period were calculated as follows:

Returned-to-shelter rate = Total Number of Dogs Returned/Total Number of Dogs Adopted.

The numerator (Total Number of Dogs Returned) represented the total amount of dogs returned-to-shelter within each subcategory of discount levels or specific campaigns during the 12-month adoption period and 6-month follow-up period, and the denominator (Total Number of Dogs Adopted) represented the total number of dogs adopted within the corresponding subcategory during the 12-month adoption period (discount categories) or specific temporary campaign period.

### 2.13. Daily Adoption Rates During Campaign and Non-Campaign Periods

Daily adoption rates were calculated as follows:

Daily Adoption Rate = Total Number of Dogs Adopted/Duration of Period (days).

The numerator (Total Number of Dogs Adopted) represented the aggregate number of adoptions during the specific campaign or non-campaign period (regardless of discount level), while the denominator (Duration of Period) was the total number of days during the specific campaign or non-campaign period.

### 2.14. Statistical Analysis

All statistical analyses were conducted in RStudio version 2024.4.2.764 [25]. A Shapiro–Wilk test for normality indicated LOS required a natural log transformation prior to analysis to meet the assumptions of normality. The effect of price discounts, breed, size, age, colour, sex, intake method (stray vs. owner surrender vs. seized), and returned (Y/N) on LOS was investigated using univariable linear regression models. Any individual terms with *p* < 0.25 at a univariable level were considered for inclusion in the multivariable model. The multivariable linear regression model was built using a backwards elimination method until all terms in the model were significant. Post hoc Tukey’s analyses were conducted to determine pairwise differences for significant terms in the final multivariable model. A *p* value < 0.05 was considered significant. For the returned-to-shelter rate analysis between different discount levels and temporary campaigns, Fisher’s exact tests were used to determine the pairwise differences.

### 2.15. Ethics Approval

Ethics approval was not necessary for this study. This research utilised only historical data provided by a council-owned animal shelter. Written permission regarding the use of the data was obtained from management at the animal shelter before commencement of the project.

## 3. Results

### 3.1. Study Population

During the 12-month period examined for adoption data (4 April 2023–3 April 2024), there were 575 rehomed entries. This total includes 16 dogs that were returned to the shelter and impounded a second time. Therefore, 559 individual dogs were rehomed during this period.

Of the 559 adopted dogs, 80 dogs were rehomed as part of ongoing price discounts: 42 dogs were sold with a pension discount (‘Pensioner Pricing’), and 38 dogs were sold as part of permanent discounts offered by the shelter (‘Long-term Special Pricing’). Both groups were excluded from analysis as they did not fit the study’s aim to investigate the effect of temporary adoption price reductions.

The final study population, therefore, included 479 dogs adopted over the 12-month period, with a mean LOS of 60.7 days (median 49 days, IQR: 29–78.5, range: 1–318). Most dogs adopted during the 12-month period had entered the facility as strays (417/479; 87.1%), with 18 (3.8%) privately surrendered and 44 (9.2%) seized. The female/male ratio within the adopted population was females 49.5% vs. males 50.5%. There was a wide range of coat colours adopted, with black dogs most adopted (138/479; 28.8%), followed by tan dogs (99/479; 20.7%) (Supplementary Table S1).

Among the 479 dogs, 14 dogs were returned to the shelter during the 6-month follow-up period (4 April 2024–3 October 2024), meaning a total of 30 dogs (6.3%) were returned to the facility over the entire 18-month study period (4 April 2023 to 3 October 2024).

### 3.2. Univariable Analysis

Discount level (*p* < 0.001), breed group (*p* < 0.0001), body size (*p* < 0.001), age group (*p* < 0.001), intake method (*p* < 0.001), and if returned (*p* = 0.009) were all significant in univariable models. Coat colour (*p* = 0.21) was not significant, however was retained in the multivariable model as *p* < 0.25, while sex (*p* = 0.51) was not significant and was excluded from the multivariable model (Supplementary Table S1).

### 3.3. Multivariable Analysis

The final multivariable model included the terms discount level, breed group, body size, age group, and intake method (all *p* < 0.05; Table 3). Return status (*p* = 0.34) and coat colour (*p* = 0.16) were not significant in the multivariable model.

#### 3.3.1. Discount Level

Dogs that did not receive a discount had a significantly shorter mean LOS (40.9 ± 2.8 days) compared to dogs with a 0–50% discount (55.1 ± 4.9 days) and dogs with a ≥75% discount (53.0 ± 3.8 days) (*p* < 0.001; Table 3). All other pairwise comparisons between discount levels were not significantly different.

#### 3.3.2. Breed Group

After controlling all other significant variables, the two breed groups that had the shortest LOS were the purebred gundog/hound/terrier group (31.5 ± 4.3 days) and the purebred and crossbred toy/non-sporting group (33.1 ± 4.3 days). These two breed groups had a significantly lower LOS compared to all other breed groups except for the crossbred gundog/hound/terrier group and purebred working dogs. Crossbred Staffordshire terriers had the longest LOS (73.0 ± 7.5 days), with crossbred working dogs having the second longest LOS with an average of 62.2 ± 5.8 days (*p* < 0.001; Table 3).

#### 3.3.3. Body Size

There was a significant difference in LOS for all body size pairwise comparisons (*p* < 0.001). Small dogs had the shortest LOS (36.2 ± 2.8 days) followed by medium dogs (40.5 ± 3.3 days) and large dogs (65.4 ± 6.9 days) (Table 3).

#### 3.3.4. Age Group

Puppies had a significantly lower LOS (38.1 ± 3.5 days) compared to all other ages except senior animals (*p* = 0.004). There was no significant difference in LOS between animals classed as young, young adult, adult, and older adult (Table 3).

#### 3.3.5. Intake Method

There was a significant difference in LOS for all intake method pairwise comparisons (*p* < 0.001). Seized dogs had the longest LOS (74.4 ± 7.4 days), privately surrendered dogs had the shortest LOS (29.9 ± 4.3 days), and stray dogs in-between (49.9 ± 2.5 days) (Table 3).

### 3.4. Animal Return

Considering the final study population after exclusions (i.e., 479 rehomed dogs rehomed over the 12-month adoption period, and 30 dogs returned-to-shelter over the entire 18-month study period), the overall effect of returned rates stratified by discount level (Table 4) and campaign type/non-campaign period (Table 5) were investigated. The overall return-to-shelter rate for the entire study population was 6.3% (30/479).

When the return rates for the different discount levels were compared, no statistically significant differences were found.

The overall return rate for temporary campaigns was 7.1% (12/169), while the return rate for the non-campaign period was 5.8% (18/310). Three of the four ‘Flash Sale’ events (Weekend Sale #1, Weekend Sale #2, and Black Friday Sale) resulted in a 0% return rate. The notable exception was Weekend Sale #3, which recorded a return rate of 41.7%. When return rates for the different campaigns were compared, the Weekend Sale #3 had a significantly higher return rate compared to all other temporary campaigns (*p* = 0.0004).

### 3.5. Effect of Temporary Campaigns on Daily Adoption Rates

Considering the final study population after exclusions (i.e., 479 rehomed dogs rehomed over the 12-month adoption period), the overall effect of temporary price discounting on daily adoption rates was investigated. In total, 169 dogs were rehomed during 90 days of the eight temporary campaigns (daily adoption rate 1.9 dogs per day). When examined further, Flash Sales (≤48 h) were the most successful temporary campaign (daily adoption rate 3.4), twice as effective as Short Campaigns (2–7 days; daily adoption rate 1.7) and almost twice as effective as Long Campaigns (>1 week; daily adoption rate 1.8). This represented an increase in daily adoptions during Flash Sales of 204% compared to non-campaign periods (daily adoption rate 1.1), and 161% compared to the overall daily adoption rate during the entire study period (daily adoption rate 1.3; Table 6).

## 4. Discussion

The primary aim of this study was to investigate a possible association between varying temporary discount levels and the LOS of dogs rehomed by an Australian municipal shelter in NSW, as well as the influence of other factors including animal signalment and intake method. The final multivariable model indicated that animals rehomed without a discount had a significantly lower LOS when compared to dogs rehomed with a 0–50% discount and ≥75% discount. There was no statistical difference between non-discounted dogs and dogs that received a 50–75% discount, and between the various discount groups (i.e., 0–50% discount vs. 50–75% discount vs. ≥75% discount). These findings suggest that discounts were likely applied reactively: highly adoptable dogs (e.g., puppies and purebreds) were rehomed quickly before price reductions were implemented, and discounts were primarily utilised for dogs that had already remained at the shelter for extended periods due to other barriers. Consequently, at least for the shelter in question, factors other than adoption price appear to be more important for adoption decisions and therefore LOS.

A secondary aim of the study was to explore any association between temporary price reductions offered during campaigns and daily adoption rates. All campaign categories (Flash Sales ≤ 48 h, Short Campaigns 2–7 days, and Long Campaigns >1 week) increased the number of daily adoptions compared to non-campaign periods when temporary price discounts were not offered. Increased rehoming rates were particularly obvious during Flash Sales: 204% higher daily adoptions compared to non-campaign periods, and 161% higher daily adoptions compared to the entire study period. Further analysis of return-to-shelter rates provided additional insights. There were no significant differences in the return rates across the various discount levels, indicating that the magnitude of the discount was not associated with owner decisions to return post-adoption. However, stratification of return rates by specific campaign revealed a significant anomaly. Notably, ‘Weekend Sale #3′ exhibited a disproportionately high return rate of 41.7%, compared to an overall return rate for dogs rehomed during temporary campaigns of 7.1%. Interestingly, this campaign also had the highest daily adoption rate (6.0 dogs/day), compared to a daily average adoption rate during temporary campaigns of 1.9 dogs/day. In other words, with this particular Flash Sale event, dogs were 3.2 times more likely to be rehomed, but 5.9 times more likely to be returned to the shelter, compared to other campaigns. This juxtaposition of a high daily adoption rate but a high return-to-shelter rate raises the possibility that this specific campaign may have facilitated impulsive decision-making by potential adopters. However, given that return rates for all the other campaigns remained low, and considering the low sample numbers (12 dogs rehomed over 2 days during ‘Weekend Sale #3′), this finding must be interpreted with caution, as it may represent a statistical outlier rather than a systemic flaw in the discount model.

Taken together, these results paint an interesting picture of what might have happened at the shelter on a micro versus a macro level. On a micro (individual) level, potential adopters made decisions based on perceived more important variables (breed, body size, and age) rather than immediate financial considerations (i.e., adoption price). On a macro (shelter) level, price discounting and associated campaign advertising presumably increased shelter exposure and the number of potential adopters visiting the shelter, thereby substantially increasing daily adoption rates. Consequently, shelters should consider both results when planning adoption campaigns, scheduling more short campaigns (≤48 h, e.g., one weekend) and fewer long campaigns, while also remembering that highly desirable dogs may not need any adoption price discount to be rehomed (i.e., avoiding campaigns when all dogs are discounted, irrespective of signalment or other factors). Instead of discounted adoption prices for these highly desirable dogs, financial resources might be better spent on post-adoption interventions, such as behavioural and medical support, to encourage adoption and reduce the likelihood of dogs being returned to the shelter following adoption.

### 4.1. ‘Reactive’ vs. ‘Proactive’ Campaigns and Their Association with Daily Adoption Rates

Many animal shelters implement ‘reactive’ adoption promotions in response to overcapacity, rather than proactively as part of a strategic plan [22]. In Australia, municipal (council) shelters operate under an open-admission policy for dogs [26]. This mandate requires them to accept any stray dog within their local government area, frequently causing facilities to reach or exceed their maximum holding capacity. Consequently, it is crucial to differentiate between animals sold during reactive promotions (i.e., those initiated due to overcapacity) and those sold through proactive promotions that involve strategic planning and advertisement. The hedonic pricing strategy employed at the shelter in this study is an example of a proactive adoption promotion, with the adoption fees for older, less desirable dogs permanently lower than those for younger, more desirable dogs to encourage the adoption of older dogs.

‘Reactive’ campaigns were observed in seven of the eight promotional campaigns run by the shelter during the 12-month adoption period, including four Flash Sale events (three ‘Weekend Sales’ and the ‘Valentine’s Day Sale’), one short campaign (the ‘Black Friday Sale’), and two long campaigns (the ‘Mad March Sale’ and the ‘New Year New Life Sale’). This reactive approach, created specifically to address overcapacity, likely meant that many dogs included in the promotions already had a prolonged LOS prior to the campaign’s commencement, leading to a significantly higher LOS in the discounted dogs compared to the more desirable, non-discounted dogs.

Of the eight promotional campaigns, ‘Staffy September’ was the only proactive campaign designed to address the over-representation and rehoming difficulty of Staffordshire terriers (both purebred and crossbred) from the shelter. Although the increase in the daily adoption rate with this proactive campaign was less pronounced than the seven reactive campaigns (Table 6), it successfully increased the daily adoption rate from 1.1 dogs/day on non-campaign days to 1.4 dogs/day during the campaign.

Irrespective of a ‘reactive’ vs ‘proactive’ strategic approach, it was clear from the current study that temporary campaigns increased the daily adoption rate while the campaign was running. The shelter promoted the campaigns in the current study via its social media accounts (Facebook and/or Instagram). This advertisement strategy aligns with industry trends; a survey by the American Society for the Prevention of Cruelty to Animals (ASPCA) indicated that 88% of shelters and rescues found Facebook had the greatest impact on increasing adoptions [27]. Furthermore, recent US-based research suggested that shelters can enhance engagement by providing more descriptive posts, specifically by including detailed information about the animal and the environment in which they are depicted [28]. Presumably, campaign advertising and media opportunities during the 12-month adoption period in the current study resulted in increased shelter exposure and visibility to members of the community contemplating dog adoption. This overall positive effect of price discounting has been previously reported by other shelters. For example, in a study carried out in Queensland, Australia, involving a free cat adoption programme, a 533% increase in the number of weekly adoptions was observed following the removal of the cost of adoption [29]. The main negative consequence that shelters need to be aware of with this promotional tactic is reduced income resulting from discounted adoption fees. Lord et al. used a computer-simulated model to report that waived and decreased adoption fees increased adoption numbers, but negatively impacted the funding and long-term sustainability of shelters [30].

### 4.2. Breed, Size, Age Group, and Intake Method as Predictors of LOS

Variables including breed, body size, age group, and intake method were found to be important predictors of LOS. In terms of breed, the shelter in the current study had an overrepresentation of Staffordshire terriers and utility/working dogs such as Australian cattle dogs, Kelpies, Bull Arabs, Siberian Huskies, and German Shepherds, similar to another shelter study in Queensland [31]. Among the different breed groups, purebred gundogs/hounds/terriers, and purebred/crossbred toy/non-sporting dogs had a significantly lower LOS compared to the other breed groups analysed, showing their desirability to potential adopters. Conversely, purebred Staffordshire terriers, crossbred Staffordshire terriers and crossbred utility/working dogs had a higher LOS and were likely less desirable among the local population of adopters. This result is similar to a research study conducted in the USA which identified terrier, hound, toy, and non-sporting breeds as having a significant positive association with successful adoption [32]. In another study at a shelter in the USA, toy and terrier breeds were less likely to be returned after adoption when compared to herding breeds (a category comparable to the working group in the Australian National Kennel Council breed standards) [33]. This suggests that toy, terrier, and non-sporting breeds have better shelter outcomes not just in Australia, but in other countries as well.

A direct relationship between dog size and LOS was observed in the current study, with the smaller the dog, the smaller the LOS. This finding aligns with prior research, with small dogs having the lowest LOS and highest adoptability, and large dogs experience the reverse [11,12,32,34]. However, one study conducted in the USA reported a slightly different pattern; while it supported the trend of smaller dogs having shorter LOS, it found that medium-sized dogs had the longest LOS [8]. Regardless, the trend that most potential adopters prefer small dogs is a consistent finding in shelters worldwide.

Puppies (<6 months) and senior dogs (>8 years) had significantly lower LOS compared to all other groups. Generally, dogs less than one year-of-age are considered highly desirable, and the younger the age, the higher the adoptability [11,14,23,34,35]. The finding that senior dogs were also deemed highly desirable by adopters has not been previously described in other literature. One possible explanation for this finding is the shelter’s protocol of collaborating with rescue partners. At the shelter, when an animal is deemed ‘difficult to rehome’ (e.g., seniors and/or dogs with concurrent diseases), rescue groups are usually contacted as soon as possible and frequently take on ownership and possession of the dog. Such transfers of ownership are conducted without a monetary fee and the animals’ subsequent outcomes are not tracked by the shelter, nor do rescue groups necessarily inform the shelter of the outcome for transferred dogs. These transfers were excluded from the definition of a successful rehoming event in the current study. The 13 senior animals retained at the shelter for direct (i.e., non-rescue group) rehoming, therefore, were likely deemed by shelter staff as easier to rehome than other senior animals due to an absence of serious co-morbidities or behavioural issues and had a lower LOS due to survivorship bias. Rescue groups also often assist the shelter by advertising these ‘difficult to rehome’ dogs using their own platforms and networks, while the dogs remain in the shelter. This external advertisement may have also increased the exposure of other non-transferred senior dogs, thereby assisting with faster adoption and a reduced LOS. Future studies should ideally include rescue collaboration data to investigate this unexpected finding for the senior age group.

Investigation of the possible impact of intake method on LOS was a highlight of the current study, given other studies have not reported on it. Privately surrendered dogs had a shorter LOS than both stray dogs and seized dogs (dogs surrendered to a council ranger or police officer). Some caution when interpreting this finding needs to be exercised due to the highly skewed population of dogs rehomed during the 12-month adoption period (<4% privately surrendered dogs). One possible explanation is that privately surrendered dogs did not undergo the mandatory holding period (7 days for microchipped animals, 14 days for non-microchipped animals). Instead, these animals became immediately available for adoption after passing medical and behavioural assessments, a process typically much shorter than the mandatory holding period. This differed to stray dogs who required a mandatory hold period before they could be assessed and become available for adoption. Additionally, privately surrendered dogs may have been more desirable to potential adopters than stray and seized dogs due to having less behavioural, medical and potential rehoming issues (e.g., house-training) noted during their assessments [10,36,37,38]. Seized dogs at the shelter are often the subject of a complaint (e.g., noise or nuisance dog), not only delaying when the animal might become available for adoption while a council or police investigation is undertaken, but also reducing their desirability to potential adopters if the complaint is upheld.

### 4.3. Limitations and Future Direction

Major limitations of the TechnologyOne data system included that it did not specify the first day that an animal became available for adoption following any mandatory hold period (stray dogs), the investigation period or type of complaint involved (seized dogs), or completion of medical and behavioural assessments (all dogs), making statistical adjustment to account for the these required administrative processes unfeasible. It also did not record medical and behavioural issues reported during the respective assessments, meaning any possible issues detailed to a potential adopter prior to adoption and affecting LOS could not be considered [20].

There was an unavoidable confounding effect between discount levels and LOS, with longer-term dogs more likely to have been part of multiple adoption campaigns and therefore multiple price discounts potentially leading to a rehoming event. This was unfortunately unable to be accounted for during statistical analysis. Limitations with the dataset also meant it was not possible to determine if adoption surges during campaign periods, particularly during Flash Sales, represented net increases or temporal shifts (i.e., adoptions that would have occurred later anyway). In the future, post-adopter surveys might be useful to help answer this question.

A larger-scale study involving multiple NSW shelters, including those with both open, limited, and closed admission policies, would provide more generalisable insights in Australia. As well as increasing statistical power with larger intake and rehoming numbers, more studies comparing outcomes across different admission types would help isolate the true effect of discounting strategies under varying operational pressures. Future research should also endeavour to utilise data capture systems capable of separating the total LOS into pre-adoption LOS (including any mandatory holding period and behavioural and medical assessments) and adoption LOS (from the date the animal first became available for adoption until adopted). Isolating the adoption phase would allow researchers to record exactly when a campaign-associated discount was first applied and analyse the combined effect of multiple campaigns for animals that were involved. Additionally, the daily adoption rate analysis did not account for seasonality, day-of-week effects, or concurrent promotional activities that might have been taking place at the shelter and impacted rehoming during both campaign and non-campaign periods. Future research should consider addressing these variables to further explore the effect of LOS associations identified in this study.

Finally, further investigations should explore the impact on rehoming of promotional campaigns that offer non-monetary incentives, such as post-adoption behavioural support. Behavioural issues often threaten the permanence of rehoming [39], and therefore ongoing support for new adopters might increase the rate of successful adoptions. In the UK, the Post Adoption Support project (launched in 2018) is designed to support individuals adopting a dog from any UK Dogs Trust Rehoming Centre via phone calls made during the first four months following adoption to provide support and referral pathways (if required). The programme has been shown to help identify medical and behavioural issues in rehomed dogs, with participation in the programme associated with improved dog retention rates at six months post-adoption [40]. Similarly, incorporating non-monetary incentives into adoption promotional campaigns, such as follow-up telephone support, at animal shelters in Australia, may boost potential owners’ confidence to adopt.

## 5. Conclusions

This study found that dogs rehomed with a ≥75% discount or a 0–50% discount had a longer LOS compared to dogs rehomed without a discounted adoption fee. This increase in LOS for discounted dogs was likely attributable to reactive adoption campaigns at the shelter in response to overcrowding, with less desirable dogs being heavily discounted after already experiencing a prolonged stay prior to price discounting. This finding suggests that other factors apart from cost, including breed, body size, age group, and intake method, are more important drivers for individual decision-making in potential adopters in NSW. However, temporary price reductions did lead to substantially higher adoption rates during targeted campaigns and therefore should be considered by shelters as an effective promotional strategy to increase overall rehoming rates. Instead of offering discounts for highly desirable dogs, shelters might consider offering additional post-adoption services to prospective owners, such as medical and behavioural support to encourage adoptions and reduce return-to-shelter rates.

## Figures and Tables

**Table 1:** Summary of the adoption fees at the open-admission municipal (council) shelter during the study period, showing the hedonic pricing strategy. Adoption prices increased during the 12-month adoption period (4 April 2023 to 3 April 2024), on 1 July 2023. Prices are given as Australian dollars (AUD).

Age Category	Adoption Fee (Full Price) (4 April 2023–30 June 2023)	Adoption Fee (Full Price) (1 July 2023–3 April 2024)
Under 4 years	AUD444.00	AUD479.00
4 to 7 years	AUD335.00	AUD362.00
Over 7 years	AUD225.00	AUD244.00

**Table 2:** Summary of eight temporary promotional campaigns and two ongoing price discount categories at the shelter studied. Temporary campaigns were further categorised by time duration: Flash Sales (≤48 h), Short Campaigns (2–7 days), and Long Campaigns (>1 week). The ongoing discounted groups (‘Long-term Special Pricing’ and ‘Pensioner Pricing’) were excluded from the study. Prices are given as Australian dollars (AUD).

Campaign	Duration	Condition	Campaign Type
Weekend Sale #1	8 April 2023–9 April 2023	All dogs sold at AUD75	Flash Sale
Weekend Sale #2	15 April 2023–16 April 2023	All dogs sold at AUD75	Flash Sale
Weekend Sale #3	27 May 2023–28 May 2023	All dogs sold at AUD50	Flash Sale
Staffy September	1 September 2023–30 September 2023	All English and American Staffordshire terriers (both purebred and crossbred) sold at AUD150	Long Campaign (species-specific [Staffordshire terriers only])
Black Friday Sale	21 November 2023–26 November 2023	Dogs under 1 year sold at AUD300, all other dogs sold at AUD200, except long-term dogs	Short Campaign (age-specific)
New Year New Life	30 December 2023–15 January 2024	All dogs sold at AUD240 except long-term dogs	Long Campaign
Valentine’s Day Sale	14 February 2024	All dogs sold at AUD100	Flash Sale
Mad March Sale	1 March 2024–31 March 2024	All dogs sold at AUD100	Long Campaign
Long-term Special Pricing	Ongoing	Dogs with length-of-stay over 120 days sold at AUD150	Ongoing Campaign (length-of-stay specific)
Pensioner Pricing	Ongoing	All dogs; adopters with valid pensioner identification receive up to 35% off the original price	Ongoing Campaign (adopter-specific)

**Table 3:** Summary of significant predictors of LOS from the multivariable model. LOS = length-of-stay, SE = standard error. Predicted means with the same superscript letter are not significantly different from each other.

Variables	*n* (%)	Mean LOS (Days)	SE	95% CI	*p* Value
Discount level					
No Discount	242 (50.5)	40.9 ^a^	2.8	31.5–42.5	
0–50% Discount	81 (16.9)	55.1 ^b^	4.9	39.3–58.6	<0.001
50–75% Discount	53 (11.1)	44.7 ^ab^	4.9	36.2–55.7	
≥75% Discount	103 (21.5)	53.0 ^b^	3.8	38.1–53.5	
Breed					
American/English Staffordshire terrier (purebred)	141 (29.4)	59.7 ^a^	4.8	51.4–70.1	<0.001
American/English Staffordshire terrier (crossbred)	53 (11.1)	73.0 ^a^	7.5	59.7–89.1	
Gundog/hound/terrier (purebred)	29 (6.1)	31.5 ^bc^	4.3	24.3–54.3	
Gundog/hound/terrier (crossbred)	84 (17.5)	42.5 ^c^	3.8	35.5–50.4	
Utility/working (purebred)	56 (11.7)	48.4 ^bc^	4.8	40.0–59.1	
Utility/working (crossbred)	90 (14.8)	62.2 ^a^	5.8	51.9–74.4	
Toy/non-sporting (purebred and crossbred)	26 (5.4)	33.1 ^ab^	4.3	25.8–42.5	
Body size					
Small (<10 kg)	197 (41.4)	36.2 ^a^	2.8	21.2–42.5	<0.001
Medium (10–30 kg)	236 (49.3)	40.5 ^b^	3.3	40.4–53.5	
Large (>30 kg)	46 (9.6)	65.4 ^c^	6.9	53.5–80.6	
Age group					
Puppies (<6 months)	157 (32.8)	38.1 ^a^	3.5	31.8–45.6	
Young (6 months to less than 12 months)	99 (20.7)	49.4 ^b^	4.3	41.7–58.6	
Young adult (1 year to less than 2 years)	98 (20.5)	53.5 ^b^	4.5	45.6–62.8	0.004
Adult (2 years to less than 4 years)	42 (8.8)	53.5 ^b^	5.1	46.5–70.1	
Older adult (4 years to less than 8 years)	70 (14.6)	56.8 ^b^	6.0	45.2–70.1	
Senior (8 years or older)	13 (2.7)	39.6 ^ab^	6.8	28.5–55.7	
Intake method					
Stray	417 (87.1)	49.9 ^b^	2.5	45.2–55.1	
Privately surrendered	18 (3.8)	29.9 ^a^	4.3	22.4–39.6	<0.001
Seized	44 (9.2)	74.4 ^c^	7.4	60.9–90.0	

**Table 4:** Summary of the returned-to-shelter rate stratified by discount level. In total, there were 30 dogs that were returned to the shelter during the 18-month study period (including the 6-month follow-up period).

Discount Level	Returned-to-Shelter (*n*)	Total Number of Dogs Sold in Each Discount Group (*n*)	Percentage of Returns Within Each Discount Group (%)
No Discount	13	242	5.4
0–50% Discount	5	81	6.2
50–75% Discount	2	53	3.8
>75% Discount	10	103	9.7
**Total**	**30**	**479**	**6.3**

**Table 5:** Summary of the returned-to-shelter rate stratified by specific campaigns. In total, there were 30 dogs that were returned to the shelter during the 18-month study period (including the 6-month follow-up period).

Campaign	Returned-to-Shelter (*n*)	Total Number of Dogs in Each Campaign (*n*)	Percentage of Returns Within Each Campaign Group (%)
Flash Sales (≤48 h)			
Valentine’s Day Sale	0	4	0
Weekend Sale #1	0	5	0
Weekend Sale #2	0	3	0
Weekend Sale #3	5	12	41.7
Short Campaigns (2–7 days)			
Black Friday Sale	0	10	0
Long Campaigns (>1 week)			
New Year New Life	0	31	0
Staffy September	2	41	4.9
Mad March Sale	5	63	7.9
Non-campaign periods	18	310	5.8
**Total**	**30**	**479**	**6.3**

**Table 6:** Summary of dogs rehomed over the 12-month adoption period, according to campaign type. Eight temporary promotional campaigns were held, further categorised by duration: Flash Sales (≤48 h), Short Campaigns (2–7 days), and Long Campaigns (>1 week). The ongoing discounted groups (‘Long-term Special Pricing’ and ‘Pensioner Pricing’) were excluded from the study.

Campaign	Duration of Campaign/Non-Campaign Period (Days)	No. Dogs Adopted (Irrespective of Discount Level)	Daily Adoption Rate (Dogs Rehomed per Day)
Flash Sales (≤48 h)			
Valentine’s Day Sale	1	4	4.0
Weekend Sale #1	2	5	2.5
Weekend Sale #2	2	3	1.5
Weekend Sale #3	2	12	6.0
**TOTAL (4 Flash Sales)**	**7 days**	**24 dogs**	**3.4 dogs/day**
Short Campaigns (2–7 days)			
Black Friday Sale	6	10	1.7
**TOTAL (1 Short Campaign)**	**6 days**	**10 dogs**	**1.7 dogs/day**
Long Campaigns (>1 week)			
New Year New Life	16	31	1.9
Staffy September	30	41	1.4
Mad March Sale	31	63	2.0
**TOTAL (3 Long Campaigns)**	**77 days**	**135 dogs**	**1.8 dogs/day**
Non-campaign periods	275	310	1.1
**TOTAL (Entire study period)**	**365 days**	**479 dogs**	**1.3 dogs/day**

## Data Availability

The data generated and analysed during the study are not publicly available. Data request should be directed to the corresponding author.

## Supplementary Materials

The following supporting information can be downloaded at: https://www.mdpi.com/article/10.3390/ani16020321/s1, Table S1: Summary of univariate analysis of factors associated with mean LOS.
