# Supplementary material for: Associations Between Adoption Discounts, Length-of-Stay, and Adoption Rates of Dogs in an Open-Admission Municipal Animal Shelter in NSW, Australia
Source: Animals (Basel). 2026 Jan 21;16(2):321. doi: 10.3390/ani16020321 (PMC12837617; doi:10.3390/ani16020321)
Supplement: Supplementary file 1 [file animals-16-00321-s001.zip › animals-4102104-supplementary.pdf]

Supplementary Table S1. Summary of univariate analysis of factors associated with mean LOS. LOS = length-of-stay, SE = standard error, CI = confidence interval.

**Variables**	**n (%)**	**Mean LOS**	**SE**	**95% CI**	***p* value**
Breed					
American/English Staffordshire terrier (purebred)	141 (29.4%)	54.2	3.15	48.4 – 60.8	
American/English Staffordshire terrier (crossbred)	53 (11.1%)	74.4	7.05	61.8 – 89.7	
Utility/working (purebred)	56 (11.7%)	48.1	4.43	40.1 – 57.6	
Utility/working (crossbred)	90 (18.8%)	52.0	3.78	45.1 – 60.0	< 0.0001
Gundog/hound/terrier (purebred)	29 (6.1%)	24.4	3.12	20.3 – 33.5	
Gundog/hound/terrier (crossbred)	84 (17.6%)	35.2	2.65	30.4 – 40.8	
Toy/non-sporting (purebred and crossbred)	26 (5.4)	31.1	4.20	23.8 – 40.5	
Body size					
Small	197 (41.1%)	33.4	1.61	30.4 – 36.7	
Medium	236 (49.3%)	56.7	2.50	52.0 – 61.9	< 0.001
Large	46 (9.6%)	79.2	7.92	65.1 – 96.4	
Intake method					
Stray	417 (87.1%)	45.6	1.61	42.5 – 48.9	
Privately surrendered	18 (3.8%)	28.3	4.79	20.3 – 39.4	< 0.001
Seized	44 (9.2%)	79.1	8.57	63.9 – 97.8	
Age group					
Puppies (< 6 months)	157 (32.3%)	36.2	2.06	32.3 – 40.4	
Young (6 months to less than 12 months)	13 (2.7%)	32.5	6.42	22.0 – 47.9	
Young adults (1 years to less than 2 years)	99 (20.7%)	47.3	3.39	41.1 – 54.5	< 0.001
Adults (2 years to less than 4 years)	70 (14.6%)	57.3	4.88	48.4 – 67.7	
Older adults (4 years to less than 8 years)	42 (8.8%)	60.7	6.68	48.9 – 75.4	
Seniors (8 years or older)	98 (20.5%)	58.6	4.22	50.9 – 67.5	
Discount level					
≥ 75% Discount	103 (21.5%)	55.5	3.96	48.2 – 63.8	
50 – 75% Discount	53 (11.1%)	50.8	5.05	41.8 – 61.8	< 0.001
0 – 50% Discount	81 (16.9%)	59.8	4.8	51.0 – 70.0	
No Discount	242 (50.5%)	39.9	1.86	36.4 – 43.7	
Returned					
No	449 (93.7%)	46.0	1.60	43.0 – 49.3	
Yes	30 (6.3%)	66.2	8.92	50.9 – 86.3	0.009
Coat colour					
Other	69 (14.4%)	36.7	3.22	30.9 – 43.6	
Tricolour	23 (4.8%)	33.9	5.14	25.1 – 45.7	
Tan	99 (20.7%)	47.8	3.50	41.4 – 55.2	
White	72 (15.0%)	45.1	3.87	38.1 – 53.3	0.21
Black	138 (28.8%)	50.7	3.14	44.9 – 57.3	
Brindle	47 (9.8%)	51.5	5.47	41.8 – 63.5	
Blue	31 (6.5%)	69.6	9.10	53.8 – 90.0	
Sex					
Female	237 (49.5%)	46.0	2.22	41.9 – 50.6	
Male	242 (50.5%)	48.2	2.30	43.9 – 52.9	0.51
